# Supplementary material for: Pharmacogenetic Influences on Individual Responses to Ocular Hypotensive Agents in Glaucoma Patients
Source: Pharmaceutics. 2025 Mar 2;17(3):325. doi: 10.3390/pharmaceutics17030325 (PMC11944811; doi:10.3390/pharmaceutics17030325)
Supplement: Supplementary file 1 [file pharmaceutics-17-00325-s001.zip › pharmaceutics-3414029-supplementary.pdf]

Table S1: Thermal cycler conditions (PCR System).

STEP		STAGE	TIME	TEMPERATURE
Initial steps		Hold	10 min	95ºC
Denature		40 cycles	15 s	95ºC
Anneal/Extend		40 cycles	60 s	60ºC

Min: minutes; s: seconds; ºC: degrees Celsius

Table S2: Post-hoc analysis of the results with statistical significance.

2A) *Baseline intraocular pressure values for PTGFR (rs3753380) in PGA group*

Group 1-2	Test Statistic	Error	Deviation of the test statistic	Significance
**HT-HM**	-8.160	13.154	0.299	0.765
**HT-WT**	17.431	13.356	1.406	0.160
**HM-WT**	-14.841	5.932	-2.502	0.012

WT: wildtype; HT: heterozygote; HM: homozygote

2B) *Rate of intraocular pressure variation values for PTGFR (rs3753380) in PGA group*

Group 1-2	Test Statistic	Error	Deviation of the test statistic	Significance
**HT-HM**	3.936	12.689	-0.643	0.520
**HT-WT**	18.777	6.057	2.878	0.004
**HM-WT**	9.271	12.440	0.745	0.456

WT: wildtype; HT: heterozygote; HM: homozygote

2C) *Baseline intraocular pressure values for PTGFR (rs3753380) in PD group*

Group 1-2	Test Statistic	Error	Deviation of the test statistic	Significance
**HT-HM**	-3.125	3.397	-0.920	0.358
**HT-WT**	7.812	3.011	2.594	0.009
**HM-WT**	4.688	2.724	1.721	0.085

WT: wildtype; HT: heterozygote; HM: homozygote

2D) *Treated intraocular pressure values for PTGFR (rs3753380) in PD group*

Group 1-2	Test Statistic	Error	Deviation of the test statistic	Significance
**HT-HM**	-3.125	3.397	-0.920	0.358
**HT-WT**	7.812	3.011	2.594	0.009
**HM-WT**	4.688	2.724	1.721	0.085

WT: wildtype; HT: heterozygote; HM: homozygote

2E) *MD values for ADRB2 (rs1042714)*

Group 1-2	Test Statistic	Error	Deviation of the test statistic	Significance
**HT-HM**	-14.564	8.837	--1.648	0.099
**HT-WT**	48.246	11.751	4.106	0.000
**HM-WT**	33.682	11.183	3.012	0.003

WT: wildtype; HT: heterozygote; HM: homozygote

***2F)** VFI values for ADRB2 (rs1042714)*

Group 1-2	Test Statistic	Error	Deviation of the test statistic	Significance
**HT-HM**	-9.180	8.713	--1.054	0.292
**HT-WT**	35.846	11.487	3.120	0.002
**HM-WT**	26.666	10.908	2.445	0.014

WT: wildtype; HT: heterozygote; HM: homozygote

***2G)** PSD values for ADRB2 (rs1042714)*

Group 1-2	Test Statistic	Error	Deviation of the test statistic	Significance
**HT-HM**	-32.993	11.183	-2.950	0.003
**HT-WT**	-42.657	11.751	-3.630	0.000
**HM-WT**	9.664	8.837	1.094	0.274

WT: wildtype; HT: heterozygote; HM: homozygote

***2H)** Baseline intraocular pressure values for ADRB2 (rs1042714)*

Group 1-2	Test Statistic	Error	Deviation of the test statistic	Significance
**HT-HM**	-4.222	8.179	-0.516	0.606
**HT-WT**	32.614	10.840	3.009	0.003
**HM-WT**	28.392	10.470	2.712	0.007

WT: wildtype; HT: heterozygote; HM: homozygote

***2I)** Rate of intraocular pressure variation values for ADRB2 (rs1042714)*

Group 1-2	Test Statistic	Error	Deviation of the test statistic	Significance
**HT-HM**	-23.305	10.222	-2.280	0.023
**HT-WT**	-28.462	10.077	-2.824	0.005
**HM-WT**	-5.156	7.809	-0.660	0.509

WT: wildtype; HT: heterozygote; HM: homozygote

***2J)** Treated intraocular pressure values among the CYP2D6 metabolizer phenotypes*

Group 1-2	Test Statistic	Error	Deviation of the test statistic	Significance
**PM-EM**	-13.190	9.877	-1.335	0.182
**PM-IM**	-15.194	9.949	-1.527	0.127
**PM-UM**	-32.500	11.167	-2.910	0.004
**EM-IM**	2.004	4.287	0.467	0.640
**EM-UM**	-19.310	6.642	-2.907	0.004
**IM-UM**	-17.310	6.747	-2.565	0.010

PM: poor metabolizer; IM: intermediate metabolizer; EM: efficient metabolizer; UM: ultrarapid metabolizer

***2K)** Rate of intraocular pressure variation values among the CYP2D6 metabolizer phenotypes*

Group 1-2	Test Statistic	Error	Deviation of the test statistic	Significance
**PM-EM**	-13.733	8.363	-1.642	0.101
**PM-IM**	-19.333	8.281	-2.335	0.02
**PM-UM**	-24.333	10.142	-2.399	0.016
**EM-IM**	5.600	3.884	1.442	0.149
**EM-UM**	-10.600	7.027	-1.509	0.131
**IM-UM**	-5.000	6.928	-0.722	0.470

PM: poor metabolizer; IM: intermediate metabolizer; EM: efficient metabolizer; UM: ultrarapid metabolizer

Table S3: Age and intraocular pressure data for the entire population (3A) and by subgroups: prostaglandin analogues (3B), beta-blockers (3C) and prostamides (3D).

3A)

	Age	bIOP	tIOP	vIOP
**Mean**	66.1	23.57	19.99	-14.04
**Median**	68	24	20	-15.59
**Standard Deviation**	12.29	4.8	4.87	23.06
**Interquartile Range**	16	7	8	-33

3B)

	Age	bIOP	tIOP	vIOP
**Mean**	66.73	23.70	20.59	-10.96
**Median**	66.00	24.00	20.00	-14.29
**Standard Deviation**	12.04	4.89	4.71	23.91
**Interquartile Range**	16.25	8.00	7.00	28.57

3C)

	Age	bIOP	tIOP	vIOP
**Mean**	63.02	23.08	18.48	-20.46
**Median**	69.00	24.00	18.00	-20.71
**Standard Deviation**	12.94	4.94	4.94	21.03
**Interquartile Range**	20.00	9.00	4.75	28.05

3D)

	Age	bIOP	tIOP	vIOP
**Mean**	71.18	24.33	20.18	-17.68
**Median**	72.00	24.00	21.00	-16.67
**Standard Deviation**	8.82	3.05	4.69	16.91
**Interquartile Range**	7.50	4.00	7.00	19.17

bIOP: baseline intraocular pressure; tIOP: treated intraocular pressure; vIOP: rate of intraocular pressure variation

Table S4: Visual field data for the entire population (4A) and by subgroups: prostaglandin analogues (4B), beta-blockers (4C) and prostamides (4D).

4A)

	MD Visual Field (dB)	VFI Visual Field (%)	PSD Visual Field (dB)
**Mean**	-4.83	88.68	4.06
**Median**	-2.25	18.97	3.44
**Standard Deviation**	7.22	97	2.4
**Interquartile Range**	5.23	9	3.36

4B)

	MD Visual Field (dB)	VFI Visual Field (%)	PSD Visual Field (dB)
**Mean**	-4.08	90.42	3.84
**Median**	-2.36	97.00	2.36
**Standard Deviation**	5.85	16.13	3.24
**Interquartile Range**	4.55	8.00	2.62

4C)

	MD Visual Field (dB)	VFI Visual Field (%)	PSD Visual Field (dB)
**Mean**	-4.76	87.82	5.45
**Median**	-1.98	97.00	2.40
**Standard Deviation**	7.33	21.12	12.10
**Interquartile Range**	5.56	8.25	3.67

4D)

	MD Visual Field (dB)	VFI Visual Field (%)	PSD Visual Field (dB)
**Mean**	-8.71	81.81	5.62
**Median**	-3.65	96.00	2.94
**Standard Deviation**	10.77	24.47	4.56
**Interquartile Range**	15.99	34.00	7.94

MD: mean deviation; VFI: visual field index; PSD: pattern standard deviation; dB: decibels

Table S5: *CYP2D6* SNP frequencies in the selected cohort and description of CNV and metabolizer phenotypes**.**

Patient (BB group)	*CYP2D6**4	*CYP2D6**41	*CYP2D6**9	*CYP2D6**2	*CYP2D6**35	CNVe9	CNVi2	CNVi6	Result Genotype	Metabolizer Phenotype
	rs3892097	rs28371725	rs5030656	rs16947	rs769258	Hs00010001_cn	Hs04083572_cn	Hs04502391_cn		
1	***1/*4**	*1/*1	*1/*1	*1/*1	*1/*1	2			*1/*4	Intermediate
2	*1/*1	*1/*1	*1/*1	***1/*2**	*1/*1	**4**	**2**	**4**	*1xN/*2xN	Ultrarapid
3	*1/*1	*1/*1	*1/*1	*1/*1	*1/*1	2			*1/*1	Efficient
4	***1/*4**	*1/*1	***1/*9**	*1/*1	*1/*1	2			*4/*9	Intermediate
5	***1/*4**	*1/*1	*1/*1	*1/*1	*1/*1	2			*1/*4	Intermediate
6	*1/*1	*1/*1	*1/*1	***1/*2**	*1/*1	**3**	**3**	**3**	*1/*2xN o *1xN/*2	Ultrarapid
7	***1/*4**	*1/*1	*1/*1	*1/*1	*1/*1	2			*1/*4	Intermediate
8	*1/*1	***1*/41**	*1/*1	***1/*2**	*1/*1	2	2	2	*1/*41	Efficient
9	*1/*1	***1/*41**	*1/*1	***2/*2**	*1/*1	2			*2/*41	Efficient
10	*1/*1	***1/*41**	*1/*1	***2/*2**	***1/*35**	2			*35/*41	Efficient
11	*1/*1	***1/*41**	*1/*1	***1/*2**	*1/*1	2			*1/*41	Efficient
12	***4/*4**	*1/*1	*1/*1	*1/*1	*1/*1	**1**	**1**	**1**	*4M/*5	Poor
13	*1/*1	*1/*1	*1/*1	***1/*2**	*1/*1	2			*1/*2	Efficient
14	*1/*1	*1/*1	*1/*1	***1/*2**	*1/*1	2			*1/*2	Efficient
15	*1/*1	***41/*41**	*1/*1	***2/*2**	*1/*1	2			*5/*41xN o *41/*41	Intermediate
16	*1/*1	*1/*1	*1/*1	***1/*2**	*1/*1	2	2	2	*1/*2	Efficient
17	***1/*4**	*1/*1	*1/*1	***1/*2**	*1/*1	2			*2/*4M	Intermediate
18	*1/*1	*1/*1	*1/*1	***2/*2**	*1/*1	2			*2/*2	Efficient
19	***1/*4**	*1/*1	*1/*1	*1/*1	*1/*1	2			*1/*4	Intermediate
20	*1/*1	*1/*1	*1/*1	*1/*1	*1/*1	**3**	**3**	**3**	*1/*1xN	Ultrarapid
21	***1/*4**	*1/*1	*1/*1	*1/*1	*1/*1	2			*1/*4	Intermediate
22	***1/*4**	***1/*41**	*1/*1	***1/*2**	*1/*1	2			*4M/*41	Intermediate
23	*1/*1	***1/*41**	*1/*1	***2/*2**	*1/*1	2			*2/*41	Efficient
24	*1/*1	*1/*1	*1/*1	*1/*1	*1/*1	2			*1/*1	Efficient
25	***1/*4**	***1/*41**	*1/*1	***1/*2**	*1/*1	2			*4M/*41	Intermediate
26	***1/*4**	*1/*1	*1/*1	***1/*2**	***1/*35**	2	2	2		Intermediate
27	*1/*1	*1/*1	*1/*1	***1/*2**	*1/*1	2	2	2	*1/*2	Efficient

BB: beta-blocker; CYP2D6: cytocrome P450 2D6; CNV: copy number variation

Table S6: Intraocular pressure and visual field parameters according to SNPs rs3766355 (A) and rs3753380 (B) of the *PTGFR* gene in PD group.

A)

PTGFR rs3766355 PD group							
**bIOP± SD**		**Kruskal-Wallis (p)**	**tIOP ± SD**	**Kruskal-Wallis (p)**	**vIOP ± SD**	**Kruskal-Wallis (p)**
**WT** (N=0)	-	p=0.393	-	p=0.300	-	p=0.268
**HT** (N=2)	25.50 ± 0.71		23.00 ± 1.41		-9.69 ± 8.05	
**HM** (N=20)	24.15 ± 3.36		19.90 ± 4.94		-18.91 ± 18.44	

**MD± SD**		**Kruskal-Wallis (p)**	**PSD ± SD**	**Kruskal-Wallis (p)**	**VFI ± SD**	**Kruskal-Wallis (p)**
**WT** (N=0)	-	p=0.732	-	p=0.775	-	p=0.507
**HT** (N=2)	-3.08 ± 2.79		5.64 ± 5.81		94.00 ± 8.49	
**HM** (N=20)	-9.28 ± 11.41		5.62 ± 4.72		80.53 ± 26.00	

B)

PTGFR rs3753380 PD group						
**bIOP± SD**		**Kruskal-Wallis (p)**	**tIOP ± SD**	**Kruskal-Wallis (p)**	**vIOP ± SD**	**Kruskal-Wallis (p)**
**WT** (N=15)	26.25 ± 2.76	p=0.021	21.33 ± 3.94	p=0.03	-15.58 ± 8.58	p=0.128
**HT** (N=3)	21.00 ± 1.73		13.33 ± 1.15		-35.94 ± 10.29	
**HM** (N=4)	23.00 ± 1.83		21.00 ± 5.77		-8.20 ± 26.89	

**MD± SD**		**Kruskal-Wallis (p)**	**PSD ± SD**	**Kruskal-Wallis (p)**	**VFI ± SD**	**Kruskal-Wallis (p)**
**WT** (N=15)	-7.46 ± 11.58	p=0.329	4.77 ± 4.67	p=0.271	89.64 ± 18.57	p=0.154
**HT** (N=3)	-14.38 ± 7.93		8.89 ± 2.64		56.33 ± 31.90	
**HM** (N=4)	-9.15 ± 11.97		6.38 ± 5.53		73.50 ± 31.47	

PTGFR: prostaglandin-F2α receptor; PD: prostamides; WT: wildtype; HT: heterozygote; HM: homozygote; bIOP: baseline intraocular pressure; tIOP: treated intraocular pressure; vIOP: rate of intraocular pressure variation; MD: medium deviation; PSD: pattern standard deviation; VFI: visual field index; N: total of eyes; p: probability value; SD: standard deviation

Table S7: Visual field parameters according to the genetic profile.

	MD± SD	Kruskal-Wallis (p)	PSD ± SD	Kruskal-Wallis (p)	VFI ± SD	Kruskal-Wallis (p)
***PTGFR* rs3766355**						
**WT** (N=6)	-2.49 ± 1.91	p=0.371	2.57 ± 0.70	p=0.584	95.67 ± 2.66	p=0.152
**HT** (N=45)	-3.39 ± 5.23		4.01 ± 3.71		93.49 ± 11.08	
**HM** (N=142)	-5.40 ± 7.83		4.15 ± 3.42		86.83 ± 20.97	
***PTGFR* rs3753380**						
**WT** (N=105)	-4.67 ± 7.45	p=0.802	3.98 ± 3.48	p=0.952	90.04 ± 17.88	p=0.600
**HT** (N=74)	-5.14 ± 7.06		4.26 ± 3.45		86.59 ± 20.49	
**HM** (N=14)	-4.30 ± 6.67		3.65 ± 3.28		89.86 ± 18.63	
***ADRB2* rs1042714**						
**WT** (N=34)	-1.22 ± 2.89	p=0.000	2.31 ± 2.11	p=0.001	93.95 ± 17.64	p=0.007
**HT** (N =67)	-6.51 ± 7.41		5.13 ± 4.09		84.35 ± 21.37	
**HM** (N=92)	-4.89 ± 7.72		3.92 ± 3.03		89.84 ± 17.09	

PTGFR: prostaglandin-F2α receptor; ADRB2: beta-2 adrenergic receptor; WT: wildtype; HT: heterozygote; HM: homozygote; MD: medium deviation; PSD: pattern standard deviation; VFI: visual field index; N: total of eyes; p: probability value; SD: standard deviation

Table S8: Intraocular pressure parameters according to the genetic profile.

	bIOP± SD	Kruskal-Wallis (p)	tIOP ± SD	Kruskal-Wallis (p)	vIOP ± SD	Kruskal-Wallis (p)
***PTGFR* rs3766355**						
**WT** (N=6)	24.00 ± 6.13	p=0.901	20.17 ± 7.41	p=0.905	-17.23 ± 16.44	p=0.320
**HT** (N=45)	23.62 ± 4.83		19.98 ± 4.45		-9.92 ± 22.58	
**HM** (N=142)	23.54 ± 4.76		19.98 ± 4.91		-15.29 ± 23.53	
***PTGFR* rs3753380**						
**WT** (N=105)	24.44 ± 4.96	p=0.040	20.03 ± 4.84	p=0.662	-18.48 ± 20.16	p=0.071
**HT** (N=74)	22.47 ± 4.44		19.86 ± 5.05		-8.16 ± 25.95	
**HM** (N=14)	23.00 ± 4.49		20.45 ± 4.25		-14.22 ± 20.42	
***ADRB2* rs1042714**						
**WT** (N=34)	25.93 ± 3.14	p=0.008	19.53 ± 3.43	p=0.291	-25.64 ± 13.84	p=0.017
**HT** (N=67)	22.88 ± 5.18		19.38 ± 5.20		-13.49 ± 22.57	
**HM** (N=92)	23.25 ± 4.77		20.65 ± 5.05		-9.90 ± 25.09	

PTGFR: prostaglandin-F2α receptor; ADRB2: beta-2 adrenergic receptor; WT: wildtype; HT: heterozygous; HM: homozygous; bIOP: baseline intraocular pressure; tIOP: treated intraocular pressure; vIOP: rate of intraocular pressure variation; N: total of eyes; p: probability value; SD: standard deviation
